# Subclinical inflammation, telomere shortening, homocysteine, vitamin B6, and mortality: the Ludwigshafen Risk and Cardiovascular Health Study

**DOI:** 10.1007/s00394-019-01993-8

**Published:** 2019-05-25

**Authors:** Irene Pusceddu, Wolfgang Herrmann, Marcus E. Kleber, Hubert Scharnagl, Michael M. Hoffmann, Brigitte M. Winklhofer-Roob, Winfried März, Markus Herrmann

**Affiliations:** 1 Department of Clinical Pathology, Hospital of Bolzano, Bolzano, Italy; 2 Department of Clinical Chemistry, University of Saarland, Homburg, Germany; 3 Medical Clinic V (Nephrology, Hypertensiology, Rheumatology, Endocrinology, Diabetology), Medical Faculty of Mannheim, University of Heidelberg, Mannheim, Germany; 4 Clinical Institute for Medical and Chemical Laboratory Diagnostics, Medical University of Graz, Auenbruggerplatz 15/1, 8036 Graz, Austria; 5 Institute for Clinical Chemistry and Laboratory Medicine, Medical Center - University of Freiburg, Faculty of Medicine, University of Freiburg, Freiburg, Germany; 6 Human Nutrition and Metabolism Research and Training Center, Institute of Molecular Biosciences, Karl-Franzens University of Graz, Graz, Austria; 7 Synlab Academy, Synlab Holding Deutschland GmbH, Mannheim, Germany

**Keywords:** Telomere length, Homocysteine, Vitamin B6, Inflammation, Mortality

## Abstract

**Purpose:**

Short telomeres and B vitamin deficiencies have been proposed as risk factors for age-related diseases and mortality that interact through oxidative stress and inflammation. However, available data to support this concept are insufficient. We aimed to investigate the predictive role of B vitamins and homocysteine (HCY) for mortality in cardiovascular patients. We explored potential relationships between HCY, B vitamins, relative telomere length (RTL), and indices of inflammation.

**Methods:**

Vitamin B6, HCY, interleukin-6 (IL-6), high-sensitive-C-reactive protein (hs-CRP), and RTL were measured in participants of the Ludwigshafen Risk and Cardiovascular Health Study. Death events were recorded over a median follow-up of 9.9 years.

**Results:**

All-cause mortality increased with higher concentrations of HCY and lower vitamin B6. Patients in the 4th quartile of HCY and vitamin B6 had hazard ratios (HR) for all-cause mortality of 2.77 (95% CI 2.28–3.37) and 0.41(95% CI 0.33–0.49), respectively, and for cardiovascular mortality of 2.78 (95% CI 2.29–3.39) and 0.40 (95% CI 0.33–0.49), respectively, compared to those in the 1st quartile. Multiple adjustments for confounders did not change these results. HCY and vitamin B6 correlated with age-corrected RTL (*r* = − 0.086, *p* < 0.001; *r* = 0.04, *p* = 0.031, respectively), IL-6 (*r* = 0.148, *p* < 0.001; *r* = − 0.249, *p* < 0.001, respectively), and hs-CRP (*r* = 0.101, *p* < 0.001; *r* = − 0.320, *p* < 0.001, respectively). Subjects with the longest telomeres had a significantly higher concentration of vitamin B6, but lower concentrations of HCY, IL-6, and hs-CRP. Multiple regression analyses identified HCY as an independent negative predictor of age-corrected RTL.

**Conclusions:**

In conclusion, hyperhomocysteinemia and vitamin B6 deficiency are risk factors for death from any cause. Hyperhomocysteinemia and vitamin B6 deficiency correlate with increased mortality. This correlation might, at least partially, be explained by accelerated telomere shortening induced by oxidative stress and systemic inflammation in these circumstances.

**Electronic supplementary material:**

The online version of this article (10.1007/s00394-019-01993-8) contains supplementary material, which is available to authorized users.

## Introduction

The accumulation of DNA damage is one of the most important factors of aging [1]. Functional telomeres and a sufficient supply with B vitamins are essential for the maintenance of genomic integrity and the prevention of premature aging [2, 3]. Vitamin B6, folate (B9), and B12 are essential co-factors in the one-carbon metabolism [4]. All three vitamins are involved in the degradation of homocysteine (HCY), which is a non-protein forming amino acid and a cytotoxic metabolite of the methionine cycle [4]. HCY is detoxified either by remethylation or by transsulfuration [4]. Prospective studies from around the world have firmly established elevated plasma HCY as a risk marker for mortality [5]. The toxic effects of HCY are at least partially mediated through oxidative damage to proteins [6] and DNA [7]. Therefore, an efficient detoxification of HCY is essential for genomic stability and cellular viability. Upon adequate availability of methionine, HCY is coupled to serine and subsequently catabolized to α-ketobutyrate and cysteine, a precursor of the principal antioxidant compound glutathione (GSH) [5]. This reaction is called transsulfuration and requires vitamin B6 as a co-factor for the enzymes cystathionine-β-synthase (CBS) and cystathionine-γ-lyase (CGL) [4]. When the exogenic supply with methionine is not sufficient, the conversion of HCY into methionine becomes activated. This reaction is called remethylation and requires 5-methyltetrahydrofolate (5-MTHF, vitamin B9) as substrate and vitamin B12 as co-factor for the methionine synthase [4]. Therefore, HCY is also considered as a functional marker of 5-MTHF and vitamin B12 availability. However, it also reflects the vitamin B6-dependent transsulfuration pathway, which transfers a sulfhydryl group from HCY to serine forming cysteine and it becomes activated in hyperhomocysteinemia (HHCY).

Telomeres are protective nucleoprotein structures at the end of all chromosomes that ensure genomic stability and prevent the loss of coding DNA. They are composed of a non-coding, repetitive DNA sequence (TTAGGG) and associated proteins [1]. Due to the inability of the DNA polymerase to fully replicate the 3′ end of chromosomes, telomeres progressively shorten with every cell division until they become critically short, lose their protective properties, and send cells into senescence, or cause cell death [1]. Telomere length in blood leucocytes has been proposed as a biomarker of biological age. Prospective observational studies in healthy and high-risk populations have shown that short telomeres substantially increase the risk of all-cause and cardiovascular (CVD) mortality [8–10]. Environmental and lifestyle factors, such as exposure to UV radiation, smoking, stress, obesity, diet, and lack of physical activity, can modify the velocity of telomere shortening [2, 11]. Many of these factors are associated with increased oxidative stress. Oxidative stress is characterized by an excess of reactive oxygen species (ROS), such as peroxides and free radicals, which assault the DNA leading to base damage, DNA strand breaks, and accelerated telomere shortening [12]. Because of their impact on the cellular redox state, HCY and vitamin B6 deficiency are potential risk factors for premature telomere shortening and accelerated cellular aging. However, existing studies are inconsistent and mainly of cross-sectional nature. For example, a negative association between telomere length and HCY was found in some studies [13–16], but not in others [17–20]. Although HCY is an established risk factor for mortality [5], little is known about the association between vitamin B6 and mortality risk [21].

Here, we investigated the predictive roles of plasma vitamin B6 and HCY concentrations for CVD and all-cause mortality in a large cohort of cardiovascular patients followed for a median period of 9.9 years. In addition, we explored potential relationships between relative telomere length (RTL), HCY, vitamin B6, and markers of systemic inflammation, such as C-reactive protein (CRP) and interleukin-6 (IL-6).

## Subjects and methods

### Study design

We analyzed baseline blood samples and clinical outcome data from 2968 Caucasian participants of the Ludwigshafen Risk and Cardiovascular Health Study (LURIC, *n* = 3316), in whom the measurements of HCY, vitamin B6, and RTL were complete. A detailed description of the LURIC study has been published previously [22]. The flowchart of the LURIC study is reported in Supplementary Fig. 1. Briefly, all patients hospitalized between June 1997 and January 2000 for diagnostic coronary angiography at the Heart Center Ludwigshafen (Germany) were enrolled in the study if they met the suitability criteria. Inclusion criteria were: German ancestry, clinical stability except for acute coronary syndromes, and the availability of a coronary angiogram. Exclusion criteria were: any acute illness other than acute coronary syndromes, any chronic disease where non-cardiac disease predominated and a history of malignancies within the past 5 years. The original aim of the present study is to investigate the associations between HCY and vitamin B6 with mortality. The secondary endpoint of the present study is to evaluate the association between HCY and B vitamins with RTL and markers of inflammation.

All patients underwent a physical examination, coronary angiography, and electrocardiography [22]. Coronary artery disease (CAD) was defined as a visible luminal narrowing of ≥ 20% stenosis in ≥ 1 of the 15 coronary segments [10, 22]. The diagnosis of myocardial infarction (MI) was either based on electrocardiographic criteria for ST elevation or non-ST elevation combined with chest pain for > 20 min (being refractory to sublingual nitrates and/or typical enzyme elevations) or based on a report of a diagnosis of MI in a medical document [10, 22]. Diabetes mellitus was diagnosed according to the 2014 criteria of the American Diabetes Association (ADA). Moreover, patients with a history of diabetes and those using oral anti-diabetics or insulin were considered diabetic [10, 22]. Alcohol intake was calculated based on information provided by the study participants at baseline in a questionnaire [22].

Information about mortality was obtained from local person registries. Two physicians blinded to participant’s baseline characteristics classified causes of death by reviewing hospital records and death certificates. In the case of disagreement about classification, the final decision was made by one of the principal investigators of LURIC after appropriate review of the data. CVD mortality was defined as death due to fatal MI, sudden cardiac death, death after cardiovascular intervention, stroke, and other causes of death due to cardiovascular diseases [10, 22]. The median follow-up time was 9.9 years (8.5–10.7).

The study was approved by the ethics committee of the Physicians Chamber of Rheinland-Pfalz and performed in accordance with the declaration of Helsinki [22]. All participants gave written informed consent [22].

### Laboratory analyses

Blood was collected in vacutainer tubes containing an anticoagulant (EDTA, citrate, or lithium heparin) or in tubes without anticoagulant. Within 30 min of venipuncture, the blood was centrifuged at 3000*g* for 10 min and immediately frozen at − 80 °C in aliquots until further analysis.

Total HCY was measured by reversed-phase high-performance liquid chromatography (HPLC) with precolumn derivatization and fluorescence detection at 470 nm (Waters, USA) [23]. Vitamin B6 and vitamin C were measured by HPLC (Waters Millennium chromatography with fluorescence detector 470, Immundiagnostik GmbH, Bensheim, Germany). Vitamin B6 results are reported in µg/L, the conversion factor to nmol/L is 4.046. Vitamin B9 concentrations were determinated using ion capture immunoassay on an AXSYM analyzer (Abbott, USA). Vitamin B9 results are reported in µg/L and the conversion factor to nmol/L is 2.266. Vitamin B12 concentrations were measured using microparticle enzyme immunoassays on an AXSYM analyzer (Abbott, USA) [23]. High-sensitive C-reactive protein (hs-CRP) was quantitated by immunonephelometry (Nephelometer II, Dade Behring, Germany) and IL-6 was measured by ELISA assay (R&D Systems Inc. USA) [23]. Plasma concentrations of tocopherols were determined by reversed-phase HPLC [24].

RTL was measured in genomic DNA using a quantitative-polymerase chain reaction (Q-PCR)-based assay, as previously reported [10]. Briefly, in each run, 40 ng of sample DNA was analyzed in duplicate, a coefficient of variation (CV) between replicates of 2.5% was considered acceptable, and the average of both replicates was calculated. When the CV between the replicates was more than 2.5%, the measurement was repeated. DNA isolated from human embryonic kidney (HEK 293, Gibco, Karlsruhe, Germany) cells was used as reference control. The PCR data were analyzed with the comparative cycle threshold (*C*_t_) method (2^−ΔΔ*C*t^) [10]. This method measures the relative expression of the telomeric sequence compared to a reference gene (relative telomere length, RTL). All Q-PCR reactions were carried out on a LightCycler Instrument (Roche). To consider the age-dependent decline of telomere length, the RTL results were corrected for age the following formula: age-corrected RTL = RTL/age [10]. Methylenetetrahydrofolate reductase (MTHFR) status was assessed in genomic DNA by PCR and HinfI digestion, as described in detail elsewhere [25].

### Statistical analyses

Non-normally distributed variables were log-transformed prior to further statistical testing. Descriptive statistics provide means (± standard deviation, SD) or medians (10–90th percentiles) for normally and non-normally distributed variables, respectively. Where indicated, quartiles of the entire study cohort were calculated. Kruskal–Wallis tests were used to identify differences between multiple groups of continuous variables. The Mann–Whitney *U* test was used to compare continuous variables between two independent groups. Chi square test and odds ratio were calculated for categorical outcomes. For the graphical presentation of results, box plots were used where the lines of each box represent the median, and the 25th and the 75th percentiles. Multiple regression analyses were performed to identify significant predictors of dependent variables (Table 3). Several models were constructed to identify the best predictor/predictors of RTL, age-corrected RTL, vitamin B6, and HCY. The independent variables included in each model are provided in the footnotes of the Table 3. Correlation analyses were performed using Spearman’s method.

The Cox proportional hazard model was used to examine the association between quartiles of vitamin B6, HCY, and time to death from any cause and from cardiovascular mortality. A crude (model 1) and adjusted models (models 2 and 3) were always performed. Model 2 included as confounders: major cardiovascular risk factors, such as sex, LDL cholesterol, HDL cholesterol, BMI, lipid lowering therapy, blood pressure, diabetes mellitus, smoking, angiographic CAD, alcohol consumption, hs-CRP, and creatinine. Model 3 included the addition vitamin C, α-tocopherol, γ-tocopherol, and MTHFR genotype.

Kaplan–Meier curves with log-rank statistics were produced to evaluate the cumulative survival during follow-up, according to quartiles of vitamin B6 or HCY.

All tests used were two-sided and *p* values < 0.05 were considered to be statistically significant. All statistical analyses were performed using SPSS (Statistical Package for the Social Sciences, version 19.0) and R v3.4.1 (http://www.r-project.org). Kaplan–Meier plots were drawn using the R-package ‘survminer’ (v5.1-1).

## Results

### Characteristics of the study population

The 2968 participants of the study, in whom the measurements of HCY, B6, and RTL were complete, had a mean age of 62.7 ± 10.6 years and 69.6% were males. Demographic, clinical, and biochemical baseline characteristics of the LURIC cohort were reported previously [15]. Table 1 summarizes patient characteristics that are relevant to the present study. While median concentrations of vitamin B6 and B9 were within the respective reference range, vitamin B12 was low normal and HCY was slightly high.

**Table 1:** Characteristics of the study cohort

Parameter	Entire cohort (*n* = 2968)	M (*n* = 2066)	F (*n* = 902)	*p* value
Anthropometric data				
M (%)	69.6	–	–	
Age (years)	63.5 (47.9–75.7)	62.5 (46.9–74.7)	65.2 (50.9–76.9)	**<** **0.001**
Physical examination				
BMI (kg/m^2^)	27.0 (22.9–32.8)	27.1 (23.4–32.5)	26.9 (21.9–33.5)	**0.015**
SBP (mmHg)	140 (112–173)	140 (112–173)	142 (110–173)	0.496
DBP (mmHg)	81 (66–96)	81 (67–97)	79 (65–94)	**<** **0.001**
Biochemical parameters				
White blood cells (× 10^3^/µL)	6.8 (4.8–9.8)	6.9 (5.0–9.9)	6.5 (4.6–9.5)	**<** **0.001**
Hemoglobin (g/dL)	13.9 (11.9–15.6)	14.3 (12.4–15.9)	13.0 (11.3–14.4)	**<** **0.001**
Glucose (mg/dL)	102 (88–154)	103 (88–152)	101 (87–159)	**0.030**
HbA1c (%)	6.0 (5.2–7.8)	6.0 (5.2–7.7)	6.0 (5.3–8.1)	**0.005**
Creatinine (mg/dL)	0.9 (0.7–1.2)	1.0 (0.8–1.2)	0.8 (0.7–1.1)	**<** **0.001**
Markers of inflammation				
hs-CRP (mg/L)	3.4 (0.7–21.5)	3.3 (0.6–22.6)	3.6 (0.7–19.6)	0.103
IL-6 (pg/mL)	3.2 (1.2–11.1)	3.3 (1.2–11.9)	3.1 (1.1–10.0)	0.051
Vitamin B and related metabolites				
Vitamin B6 (µg/L)	8.9 (3.5–22.3)	9.3 (3.8–22.9)	8.0 (3.1–20.7)	**<** **0.001**
Vitamin B6 < 5 µg/L (%)	21	19	25	
Vitamin B12 (pmol/L)	344 (197–636)	340 (197–616)	354 (200–692)	**0.044**
Vitamin B9 (µg/L)	7.8 (4.7–12.0)	7.6 (4.6–11.8)	8.2 (5.0–12.4)	**<** **0.001**
HCY (µmol/L)	12.3 (8.1–19.8)	12.6 (8.4–19.8)	11.5 (7.5–19.6)	**<** **0.001**
HCY < 12 µmol/L (%)	48	45	55	
Relative telomere length				
RTL	1.79 (0.46–4.95)	1.76 (0.46–4.90)	1.86 (0.46–5.11)	0.564
Age-corrected RTL	0.0280 (0.0070–0.0865)	0.0280 (0.0071–0.0877)	0.0279 (0.0069–0.0841)	0.428

Variables are reported as mean ± SD or median (10–90th percentiles) according to the normal and non-normal distribution of values, respectively. Statistically significant differences are reported in bold

### Vitamin B6, HCY, and mortality

**Fig. 1:**
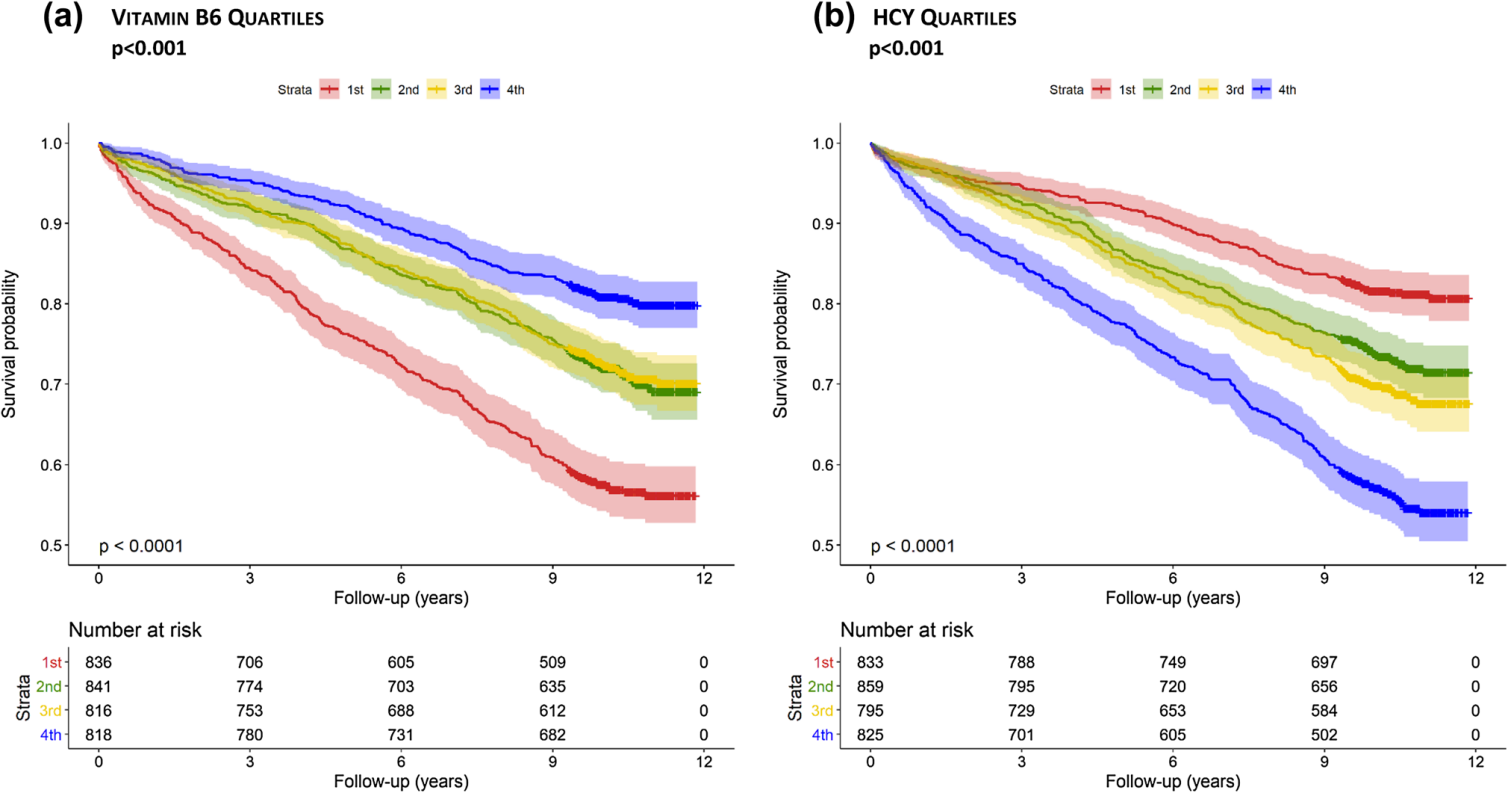
Kaplan–Meier plots. Cumulative survival according to quartiles of HCY (**a**); vitamin B6 (**b**)

During a median follow-up period of 9.9 years, 2068 patients stayed alive and 900 died. Plasma vitamin B6 was identified as a strong independent predictor for all-cause mortality. The Kaplan–Meier curve showed a continuous increase in mortality with decreasing concentrations of vitamin B6 (Fig. 1a). Cox-regression analysis confirmed vitamin B6 as a significant predictor for all-cause and CVD mortality (Table 2, model 1). In the crude model, subjects with the highest vitamin B6 concentrations (4th quartile) had 59% lower risk to die during follow-up, compared to those in the 1st quartile. This effect remained highly significant after adjustment for common confounders (Table 2, model 2), sex, LDL cholesterol, HDL cholesterol, BMI, lipid lowering therapy, blood pressure, diabetes mellitus, smoking, CAD, alcohol consumption, hs-CRP, and creatinine.

**Table 2:** Cox proportional hazard models for all-cause and CVD mortality according to HCY and vitamin B6 quartiles

All-cause mortality	*N*° deceased (%)/alive (%) patients	Model 1 HR (95%)	*p* value	Model 2 HR (95%)	*p* value	Model 3 HR (95%)	*p* value
HCY quartiles (*n* = 2968)							
1st (< 9.8 µmol/L, *n* = 756)	146 (19)/610 (81)	Ref.		Ref.		Ref.	
2nd (9.9–12.4 µmol/L, *n* = 774)	210 (27)/564 (73)	1.48 (1.20–1.83)	**<** **0.001**	1.28 (1.04–1.58)	**0.022**	1.43 (1.12–1.82)	**0.004**
3rd (12.5–15.5 µmol/L, *n* = 701)	217 (31)/484 (69)	1.73 (1.40–2.13)	**<** **0.001**	1.44 (1.16–1.78)	**0.001**	1.49 (1.16–1.90)	**0.002**
4th (> 15.6 µmol/L, *n* = 737)	327 (44)/410 (56)	2.77 (2.28–3.37)	**<** **0.001**	2.03 (1.66–2.49)	**<** **0.001**	2.13 (1.69–2.70)	**<** **0.001**
Vitamin B6 quartiles (*n* = 2968)							
1st (< 5.6 µg/L, *n* = 742)	312 (42)/430 (58)	Ref.		Ref.		Ref.	
2nd (5.7–8.9 µg/L, *n* = 760)	223 (29)/537 (71)	0.62 (0.52–0.73)	**<** **0.001**	0.72 (0.61–0.86)	**<** **0.001**	0.70 (0.57–0.85)	**<** **0.001**
3rd (9.0–14.1 µg/L, *n* = 719)	213 (30)/506 (70)	0.62 (0.52–0.74)	**<** **0.001**	0.75 (0.62–0.90)	**0.002**	0.75 (0.61–0.93)	**0.008**
4th (> 14.2 µg/L, *n* = 747)	152 (20)/595 (80)	0.41 (0.33–0.49)	**<** **0.001**	0.54 (0.44–0.67)	**<** **0.001**	0.55 (0.44–0.70)	**<** **0.001**

CVD mortality	Model 1 HR (95%)	*p* value	Model 2 HR (95%)	*p* value	Model 3 HR (95%)	*p* value
HCY quartiles						
1st (< 9.8 µmol/L, *n* = 756)	Ref.		Ref.		Ref.	
2nd (9.9–12.4 µmol/L, *n* = 774)	1.50 (1.21–1.86)	**<** **0.001**	1.30 (1.05–1.61)	**0.017**	1.46 (1.14–1.87)	**0.002**
3rd (12.5–15.5 µmol/L, *n* = 701)	1.74 (1.41–2.16)	**<** **0.001**	1.45 (1.17–1.79)	**0.001**	1.50 (1.16–1.92)	**0.002**
4th (> 15.6 µmol/L, *n* = 737)	2.78 (2.29–3.39)	**<** **0.001**	2.04 (1.66–2.51)	**<** **0.001**	2.16 (1.71–2.74)	**<** **0.001**
Vitamin B6 quartiles						
1st (< 5.6 µg/L, *n* = 742)	Ref.		Ref.		Ref.	
2nd (5.7–8.9 µg/L, *n* = 760)	0.62 (0.52–0.74)	**<** **0.001**	0.72 (0.60–0.86)	**<** **0.001**	0.70 (0.57–0.85)	**<** **0.001**
3rd (9.0–14.1 µg/L, *n* = 719)	0.63 (0.53–0.75)	**<** **0.001**	0.75 (0.63–0.91)	**0.003**	0.76 (0.61–0.93)	**0.001**
4th (> 14.2 µg/L, *n* = 747)	0.40 (0.33–0.49)	**<** **0.001**	0.54 (0.44–0.67)	**<** **0.001**	0.55 (0.43–0.70)	**<** **0.001**

Statistically significant *p* values are reported in bold

*Model 1* crude model, *Model 2* adjusted for sex, *LDL* cholesterol, *HDL* cholesterol, *BMI* lipid lowering therapy, blood pressure, diabetes mellitus, smoking, *CAD* alcohol consumption, *hs-CRP* and creatinine, *Model 3* in addition adjusted for MTHFR genotype, vitamin C, α-tocopherol, and γ-tocopherol, *Ref.* reference

In view of the functional relationship between vitamin B6 and HCY, the next step was to examine the relationship between plasma HCY and mortality. Subjects with the lowest baseline plasma HCY concentrations (1st quartile) had the lowest all-cause mortality compared to those with higher HCY concentrations (*p* < 0.001; Fig. 1b). Cox-regression analysis showed a continuous increase in mortality with increasing quartiles of baseline HCY concentrations (Table 2). Compared to the lowest quartile, the unadjusted hazard ratios for death from all causes and from CVD mortality in the fourth HCY quartile was nearly three times higher (Table 2, model 1). This effect remained highly significant after adjustment for common confounders (Table 2, model 2), sex, LDL cholesterol, HDL cholesterol, BMI, lipid lowering therapy, blood pressure, diabetes mellitus, smoking, CAD, alcohol consumption, hs-CRP, and creatinine.

As HCY levels are influenced by the genetic variants of the MTHFR enzyme, we added the MTHFR genotype as co-variate in the Cox-regression analyses. In this model HCY and vitamin B6 remained highly significant predictors for all-cause mortality and CVD mortality even with further adjustment for MTHFR genotype (Table 2, model 3).

Considering that other micronutrients, vitamin C, α-tocopherol and γ-tocopherol may also influence oxidative processes and ultimately impact mortality rates, we calculated an additional model adjusted for these variables. In this model HCY and vitamin B6 remained highly significant predictors for all-cause mortality and CVD mortality even with further adjustment for vitamin C, α-tocopherol, and γ-tocopherol (Table 2, model 3).

Acute coronary syndrome (ACS) is another important cause of inflammation. As 31% of the study population suffered from ACS, we performed separated regression analyses for patients with and without ACS (Suppl. Table 1). HCY and vitamin B6 were a significant predictor for all-cause mortality in both groups.

### Vitamin B6, HCY, and telomere biology

**Fig. 2:**
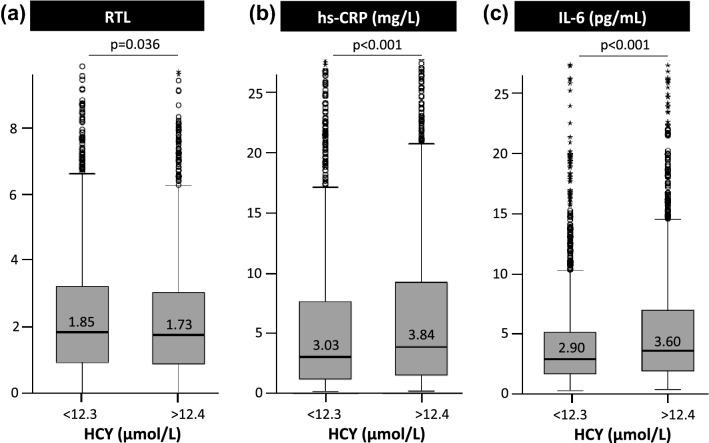
RTL (**a**), hs-CRP (**b**), and IL-6 **c** in subjects with HCY below or above HCY the cohort median of 12.3 μmol/L

Age-corrected RTL and crude RTL were significantly correlated with HCY (*r* = − 0.086; *p* < 0.001 and *r* = − 0.044; *p* = 0.016, respectively). Furthermore, age-corrected RTL was significantly correlated with vitamin B6 (*r* = 0.04; *p* = 0.029). When compared to all other participants, subjects with the longest telomeres (4th quartile of age-corrected RTL) had a higher median concentration of vitamin B6 and lower concentrations of plasma HCY (Table 3). In addition, RTL was greater in subjects with plasma HCY concentrations below the cut-off of 12 μmol/L when compared to those above (*p* = 0.036, Fig. 2a). On the other side, age-corrected RTL was slightly greater in subjects with vitamin B6 above the median of 8.9 µg/L compared to those below, although the difference was not statistically significant (age-corrected RTL = 0.0274 vs 0.0287; *p* = 0.072).

**Table 3:** Vitamin B status and markers of inflammation according to age-corrected RTL and RTL quartiles

Age-corrected RTL quartiles	1st (*n* = 739) < 0.0140	2nd (*n* = 748) 0.0141–0.0280	3rd (*n* = 738) 0.0281–0.0509	4th (*n* = 743) > 0.0510	*p* value	*p* value 1–3 vs 4
HCY (µmol/L)	12.8 (8.5–20.1)	12.4 (8.1–19.9)	12.2 (7.9–21.2)	11.7 (7.8–18.6)	**<** **0.001**	**<** **0.001**
	12.5 (8.1–20.3)					
Vitamin B6 (µg/L)	8.8 (3.7–21.7)	8.6 (3.4–22.4)	8.4 (3.4–21.7)	9.7 (3.6–23.6)	**0.004**	**0.001**
	8.6 (3.5–21.9)					
hs-CRP (mg/L)	3.19 (0.66–21.66)	3.54 (0.71–24.20)	3.55 (0.73–20.60)	3.05 (0.53–20.25)	0.067	**0.017**
	3.43 (0.70–21.90)					
IL-6 (pg/mL)	3.34 (1.23–11.50)	3.43 (1.22–11.95)	3.26 (1.17–11.59)	2.82 (1.07–9.87)	**0.001**	**<** **0.001**
	3.34 (1.21–11.59)					

RTL quartiles	1st (*n* = 739) < 0.8915	2nd (*n* = 748) 0.8916–1.7881	3rd (*n *= 738) 1.7882–3.1088	4th (*n* = 743) > 3.1089	*p* value	*p* value 1–3 vs 4
HCY (µmol/L)	12.6 (8.3–19.6)	12.3 (8.14–19.7)	12.2 (7.8–20.7)	12.0 (8.1–19.1)	0.085	0.298
	12.4 (8.1–20.0)					
Vitamin B6 (µg/L)	9.0 (3.8–22.0)	8.6 (3.3–21.7)	8.7 (3.5–22.0)	9.4 (3.4–23.6)	0.244	0.328
	8.7 (3.5–21.9)					
hs-CRP (mg/L)	3.08 (0.66–21.87)	3.47 (0.66–22.90)	3.72 (0.74–19.67)	3.06 (0.59–21.50)	0.110	0.166
	3.43 (0.69–21.60)					
IL-6 (pg/mL)	3.31 (1.21–11.47)	3.32 (1.15–11.21)	3.33 (1.26–11.16)	2.91 (1.10–10.72)	**0.026**	**0.001**
	3.32 (1.21–11.29)					

Variables are reported as median (10–90th percentiles). Statistically significant differences are reported in bold

In subjects with plasma HCY below 12 μmol/L, the odds ratio for highest age-corrected RTL (4th quartile age-corrected RTL > 0.0510) is 1.23 (95% CI 1.11–1.37; *p* < 0.001).

### Vitamin B6, HCY, telomere biology, and markers of inflammation

IL-6 and hs-CRP were significantly lower in subjects with the longest telomeres (4th quartile of age-corrected RTL) compared to subjects with the shortest telomeres (1st quartile of age-corrected RTL; Table 3). In addition, IL-6 and hs-CRP showed significant inverse correlation with vitamin B6 (*r* = − 0.249; *p* < 0.001 and *r* = − 0.320; *p* < 0.001, respectively) but positive correlation with HCY (*r* = 0.148; *p* < 0.001 and *r* = 0.101; *p* < 0.001, respectively). Hs-CRP and IL-6 were higher in subjects with plasma HCY concentrations above the median of 12.3 μmol/L when compared to those below (Fig. 2b, c). Furthermore, hs-CRP and IL-6 were significantly higher in subjects in the 1st quartile of vitamin B6 compared to all the other subjects (Suppl. Table 1). Conversely, subjects in the 1st quartile of HCY had significantly lower levels of hs-CRP and IL-6 compared to all the other subjects (Suppl. Table 2).

Multiple backward regression analyses identified HCY and IL-6 as significant independent predictors of age-corrected RTL (Table 4B). In addition, HCY, IL-6, hs-CRP, α-tocopherol, γ-tocopherol, and vitamin C were independent predictors of vitamin B6 (Table 4C). Finally, IL-6, MTHFR genotype, age-corrected RTL, and vitamin B6 were independent predictors of HCY (Table 4D).

**Table 4:** Summary of the multiple regression analyses

Predictor variables	Unstandardized *B*	95% CI for *B*	Standardized *β*	*t*	*p* value
A) Dependent variable: RTL					
Age	− 0.004	− 0.005 to − 0.002	− 0.084	− 3.954	< 0.001
B) Dependent variable: age-corrected RTL					
HCY	− 0.187	− 0.311 to − 0.063	− 0.063	− 2.960	0.003
IL-6	− 0.048	− 0.101 to − 0.004	− 0.039	− 1.821	0.069
C) Dependent variable: vitamin B6					
HCY	− 0.301	− 0.378 to − 0.224	− 0.152	− 7.644	**<** **0.001**
IL-6	− 0.051	− 0.090 to − 0.011	− 0.061	− 2.536	**0.011**
hs-CRP	− 0.133	− 0.159 to − 0.107	− 0.240	− 10.084	**<** **0.001**
α-Tocopherol	0.004	0.003–0.005	0.144	7.270	**<** **0.001**
γ-Tocopherol	− 0.036	− 0.049 to − 0.022	− 0.102	− 5.150	**<** **0.001**
Vitamin C	− 1.471E−7	0.000–0.000	− 0.043	− 2.205	**0.028**
D) Dependent variable: HCY					
IL-6	0.039	0.022–0.057	0.093	4.361	**<** **0.001**
MTHFR genotype	0.013	0.004–0.023	0.057	2.764	**0.006**
Age-corrected RTL	− 0.020	− 0.033 to − 0.006	− 0.058	− 2.786	**0.005**
Vitamin B6	− 0.083	− 0.104 to − 0.062	− 0.164	− 7.699	**<** **0.001**

Regression analyses were performed by backward variable selection. All variables were log-transformed. Predictors included in the models are: A) age, vitamin B6, HCY, IL-6, hs-CRP, MTHFR genotype, α-tocopherol, γ-tocopherol, and vitamin C; B) vitamin B6, HCY, IL-6, hs-CRP, MTHFR genotype, α-tocopherol, γ-tocopherol, and vitamin C; C) age-corrected RTL, HCY, IL-6, hs-CRP, MTHFR genotype, α-tocopherol, γ-tocopherol, and vitamin C; D) age-corrected RTL, vitamin B6, IL-6, hs-CRP, MTHFR genotype, α-tocopherol, γ-tocopherol, and vitamin C

## Discussion

We show that vitamin B6 and HCY are independent risk factors for all-cause mortality in cardiovascular patients. Relationships of both analytes with RTL suggest that vitamin B6 deficiency and HHCY trigger genomic aging and ultimately accelerate biological aging of the entire organism. Significant associations between HCY, RTL, and inflammatory biomarkers, such as IL-6, point to systemic inflammation and oxidative stress as potential mechanisms that could explain how vitamin B6 deficiency and HHCY accelerate telomere shortening and increase mortality.

### HCY, RTL, and mortality

HHCY [4, 5] and low RTL [8, 10] are established risk factors for all-cause and cardiovascular mortality. An earlier study from our group in the LURIC cohort has shown that RTL predicts mortality [10]. The present results expand these data, demonstrating that HCY is another independent predictor of mortality in LURIC. In this study population, median plasma HCY concentration was 12.3 µmol/L, which is slightly above the cut-off of 12 µmol/L, recommended by the German, Austrian, and Swiss Homocysteine Society (DACH-Liga homocysteine) [26]. Subjects with an HCY concentration of 9.8 µmol/L or higher had a 28% higher risk to die from any cause or CVD compared to those with lower HCY. In addition, subjects with shorter telomeres (1st quartile) were characterized by higher levels of HCY.

Together with existing data, our findings raise the question if there is a direct link between HCY and RTL. The previous studies support an inverse relation between HCY and RTL [13–19]. For example, Richards et al. report an 111 base-pair difference in RTL between the highest and lowest tertile of plasma HCY corresponding to 6 years of telomeric aging [13]. Zhang et al. showed lower human telomerase reverse transcriptase (hTERT) mRNA levels and a reduced methylation of the hTERT promotor region in hyperhomocysteinemia [27]. The same group confirmed a mechanistic link between HCY and telomeres in an in vivo study using hyperhomocysteinemic mice [28]. The leucocytes of these animals were characterized by lower amounts of hTERT mRNA and reduced methylation of the hTERT promotor region [28]. Besides telomere shortening, HHCY also causes other types of DNA damage, such as the formation of micronuclei [29] and DNA interstrand cross-links [7]. DNA hypomethylation is another potential mechanism that accelerates telomere shortening. In an earlier study, 1 year of vitamin B6, B12, B9, and D supplementation lowered HCY and altered long interspersed nuclear elements (LINE-1) methylation, a surrogate marker of global DNA methylation [30]. At the end of treatment, hyperhomocysteinemic subjects who were simultaneously treated with B and D vitamins had longer telomeres than those supplemented with vitamin D only [30]. Furthermore, LINE 1 methylation status was correlated with RTL [30].

### Vitamin B6 and mortality

The present study revealed a strong inverse relationship between plasma vitamin B6 and mortality. Subjects with vitamin B6 levels above 14.2 µg/L had a 59% lower risk to die from all-cause or CVD compared to those with lower vitamin B6 concentrations. In addition, shorter telomeres (1st quartile) had lower levels of vitamin B6 compared to those with longer telomeres. Until today, only a few large studies have investigated vitamin B6 for mortality [21, 31]. Patterson et al. measured 28 biomarkers in 1911 middle aged men from the Caerphilly Prospective Study (CaPS study), and found an inverse relationship between vitamin B6 and non-CVD mortality (HR 0.83; 95% CI 0.75–0.93; *p* < 0.01) [31]. However, this association was not present for CVD mortality. The epidemiologic nature of this study implies a lower mortality risk and explains at least partially the smaller effect size [31]. Although the results of CaPS and LURIC are concordant, it should be mentioned that, in CaPS, the causes of death were not verified as rigorously as in LURIC. Therefore, it is not surprising that, in LURIC, vitamin B6 was also associated with CVD mortality. In another study, Ulvik et al. analyzed four markers of vitamin B6 status and metabolism in 7913 patients with stable angina pectoris or MI from two cohorts [21]. After adjustment for common confounders, plasma vitamin B6 (pyridoxal phosphate) predicted all-cause mortality in MI patients [21]. However, after inclusion of inflammatory markers into the Cox-regression model, this association was no longer significant [21]. The pyridoxic acid/(pyridoxal + pyridoxyl phosphate) ratio, a marker of vitamin B6 catabolism, was positively associated with all-cause mortality [21]. The authors hypothesized that low plasma vitamin B6 concentrations may be secondary to inflammatory activation [21]. In addition, they suggested that the inflammatory process, rather than low vitamin B6, is responsible for the relation with all-cause mortality [21]. However, this theory is in contrast to the present results that identified vitamin B6 as an independent predictor of all-cause mortality after adjustment for hs-CRP, IL-6, and other established risk factors.

Our study also showed a significant correlation between vitamin B6 and RTL. In the highest quartile of age-corrected RTL, vitamin B6 was significantly higher than in the other three quartiles together. These results are in agreement with a very recent study that demonstrated a positive relationship between vitamin B6 intake and telomere length in 10,568 participants of the National Health and Nutrition Examination Survey (NHANES) [32]. In contrast, other studies found no significant relationship between RTL and plasma vitamin B6 or vitamin B6 intake [17–20].

### Mechanisms

The present results point towards oxidative stress and chronic inflammation as the central mechanism that link HCY, vitamin B6, and RTL with mortality. Individuals with the longest telomeres (4th quartile) had significantly lower concentrations of HCY, IL-6, and hs-CRP, but higher vitamin B6. Backward regression analysis identified HCY and IL-6 as the two strong determinants of RTL. High plasma concentrations of HCY disrupt enzymatic and non-enzymatic antioxidant defense mechanisms in many tissue types including myocardium, liver, and brain [33–35]. The resulting oxidative stress causes intracellular and extracellular damage and promotes inflammation [36]. Interestingly, the relationship between HCY and chronic inflammation is not just a one-way road. In vitro experiments have shown that pro-inflammatory cytokines, such as interleukin-1β (IL-1β) and TNF-alpha, alter the cells’ redox state and increase the extracellular HCY concentration in a concentration-dependent fashion [35]. Moreover, systemic inflammation increases vitamin B6 catabolism and cellular uptake, resulting in reduced vitamin B6 plasma concentrations [37]. An intervention study by Ulvik et al. suggests that pyridoxine treatment improves systemic inflammation in patients with SAP [38]. Considering these results in conjunction with vitamin B6’ role as a co-factor of CBS and CGL, we believe that vitamin B6 represents an important link between HCY, the cells’ redox state, and systemic inflammation.

**Fig. 3:**
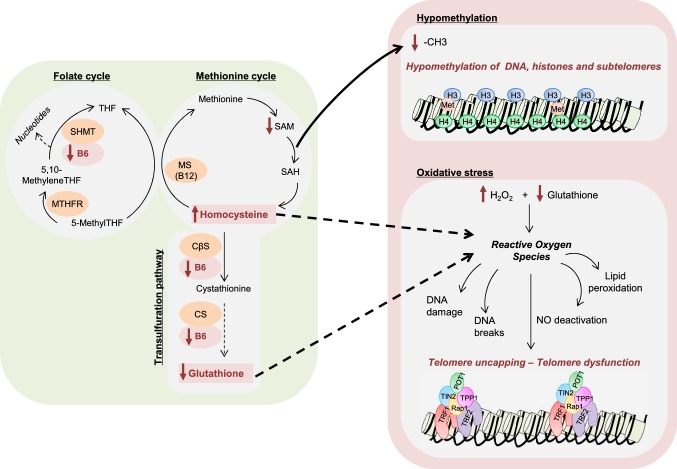
Key mechanisms underlying the relationship between B vitamins, homocysteine, and telomere function. Decreased vitamin B12 concentrations impair the remethylation of homocysteine to methionine through the methionine synthase, which is vitamin B12-dependent. MTHFR catalyzes the irreversible reduction of 5,10-methyleneTHF to 5-methylTHF, which is the methyl donor required for the conversion of homocysteine to methionine via methionine synthase. 5,10-MethyleneTHF can be regenerated from tetrahydrofolate produced in the methionine synthase reaction by the donation of a methyl group from the amino acid serine in a reaction catalyzed by SHMY, a vitamin B6-dependent enzyme. In addition, the methyl group from 5,10-methyleneTHF is used for the production of nucleotides. Hyperhomocysteinemia reduces the production of SAM, the universal methyl-donor group. The consequent reduced –CH_3_ availability impairs the methylation of DNA, subtelomeric regions, and proteins such as histones. Deficiency of vitamin B6 leads to impaired conversion of HCY to cystathionine, leading ultimately to decreased production of glutathione. Hyperhomocysteinemia and reduced glutathione induce the formation of reactive oxygen species that induce DNA damage, DNA breaks, NO deactivation, lipid peroxidation, and telomere uncapping leading to telomere dysfunction. *CβS* cystathionine-beta-synthase, *CS* cystathionase, *H* histone, *Met* methylated, *MS* methionine synthase, *MTHFR* methylenetetrahydrofolate reductase, *NO* nitric oxide, *POT1* protection of telomeres 1, *RAP1* repressor/activator protein 1, *SAM* S-adenosyl methionine, *SAH* S-adenosyl homocysteine, *SHMT* serine hydroxymethyl transferase, *THF* tetrahydrofolate, *TIN2* TRF1 interacting protein 1, *TPP1* TINT1/PIP1/PTOP 1, *TRF1* telomere repeat binding factor 1, *TRF2* telomere repeat binding factor 2

The previous studies have proposed a mechanistic link between oxidative stress, systemic inflammation, and telomere attrition [39, 40]. Activation of the complement system and an increased formation of ROS, such as superoxide, hydrogen peroxide, and others, are key components of inflammation that damage telomeric DNA [12]. In a cross-sectional study from our group advanced glycation end products (AGEs), a surrogate marker of oxidative stress correlated inversely with RTL (not published data). Although oxidative stress-induced telomere shortening is not fully understood, several mechanisms seem to be involved (Fig. 3). Formation of 8-oxoguanine is one of the most common DNA damages resulting from ROS and is responsible for the mismatch pairing with adenine leading to G-to-T and C-to-A substitutions in the genome [12]. G-rich telomeres are particular sensitive to these substitutions. In addition, ROS impair the activity of endonuclease III-like protein 1, which is involved in the repair of oxidative DNA damage [41]. In vitro studies have shown that oxidative DNA damage impairs recognition and binding of the shelterin proteins telomeric repeat binding factor 1 (TRF1) and telomeric repeat binding factor 2 (TRF2) to telomeric DNA [42]. Ultimately, the different forms of oxidative DNA damage compromise the protective function of telomeres and trigger systemic inflammation and cellular senescence through a senescence-associated secretory phenotype (SASP) [1]. Of course, systemic inflammation and oxidative stress do not act in isolation. Elevated HCY concentrations are associated with hypomethylation of proteins and DNA [43]. Hypomethylation leads to altered gene expression and impairs genomic integrity [44]. Methylation of DNA promotor regions modifies gene expression and, thus, contributes to disease development [45]. Furthermore, HCY-related hypomethylation affects methylation status of telomeric and subtelomeric region and also influences the gene intron region, which often becomes activated in hypomethylation [43]. In a supplementation study with B and D vitamins on elderly subjects over a period of 1 year, we could show that vitamin supplementation significantly influenced the correlation between RTL and B vitamin metabolites (methyl group metabolism) but also the relation between LINE1-methylation as surrogate marker for total DNA methylation and HCY [30].

### Strengths and limitations

The major strength of the present study is the large-scale prospective nature of the LURIC study. We speculate that vitamin B6 could affect telomere length through altered DNA or protein methylation, but epigenetic analyses have not been completed in the LURIC study.

Furthermore, vitamin B6, B12, and B9 are functionally linked in the one-carbon metabolism. As deficiencies of one or more of these micronutrients contribute to the development of HHCY, the actual relevance of vitamin B6 to telomere length is difficult to determine. In our large epidemiological study, not only vitamin B6 and HCY were significant predictors of all-cause and CVD mortality. Associations between mortality and vitamin B9 or vitamin B12 were also seen, but were weaker than for vitamin B6 or HCY. For vitamin B12, we found a U-shaped association. It should also be mentioned that only HCY and vitamin B6 correlated with RTL, but not vitamin B9 nor vitamin B12. This may indicate different pathomechanisms for B vitamin deficiencies in relation to mortality. Nevertheless, the involvement of vitamin B6 in GSH production, the principal antioxidant compound, led us to analyze the impact of inflammation on the relationship between vitamin B6 and telomere length.

The question if vitamin B6 supplementation could improve telomere length and survival cannot be answered due to the design of our study.

## Conclusion

We conclude that high plasma homocysteine and low vitamin B6 concentrations are risk markers for death from any cause. In addition, our results suggest that HHCY and vitamin B6 deficiency increase mortality through accelerated telomere shortening caused by oxidative stress and systemic inflammation. Despite a strong statistical power of the LURIC study, future observational and intervention studies should confirm our results and explore if vitamin B6 supplementation can slow down telomere shortening and prevent premature cellular aging.

## Electronic supplementary material

Below is the link to the electronic supplementary material.

Supplementary material 1 (DOCX 33 kb)
