# Supplementary material for: Subclinical inflammation, telomere shortening, homocysteine, vitamin B6, and mortality: the Ludwigshafen Risk and Cardiovascular Health Study
Source: Eur J Nutr. 2019 May 25;59(4):1399–411. doi: 10.1007/s00394-019-01993-8 (PMC7230054; doi:10.1007/s00394-019-01993-8)
Supplement: Supplementary file 1 — Supplementary material 1 (DOCX 33 kb) [file 394_2019_1993_MOESM1_ESM.docx]

**Supplementary Data**

| Suppl. Table 1: Cox proportional hazard models for all-cause mortality according to HCY and vitamin B6 quartiles in patients affected and non-affected by ACS. | | |
| --- | --- | --- |
|  |  |  |
| Quartiles | Model 1  HR (95%) | P-Value |
| **ACS patients (n=1036)** | | |
| **HCY quartiles** |  |  |
| 1^st^ (<9.8 µmol/L) | Ref. |  |
| 2^nd^ (9.9-12.4 µmol/L) | 1.22 (0.86-1.73) | 0.267 |
| 3^rd^ (12.5-15.5 µmol/L) | 1.18 (0.83-1.70) | 0.349 |
| 4^th^ (>15.6 µmol/L) | 1.84 (1.30-2.62) | **0.001** |
| **Vitamin B6 quartiles** |  |  |
| 1^st^ (<5.6 µg/L) | Ref. |  |
| 2^nd^ (5.7-8.9 µg/L) | 0.72 (0.54-0.96) | **0.026** |
| 3^rd^ (9.0-14.1 µg/L) | 0.63 (0.47-0.86) | **0.003** |
| 4^th^ (>14.2 µg/L) | 0.49 (0.34-0.70) | **<0.001** |
| **Non- ACS patients (n=2280)** | | |
| **HCY quartiles** |  |  |
| 1^st^ (<9.8 µmol/L) | Ref. |  |
| 2^nd^ (9.9-12.4 µmol/L) | 1.37 (1.06-1.76) | **0.016** |
| 3^rd^ (12.5-15.5 µmol/L) | 1.71 (1.33-2.20) | **<0.001** |
| 4^th^ (>15.6 µmol/L) | 2.25 (1.78-2.86) | **<0.001** |
| **Vitamin B6 quartiles** |  |  |
| 1^st^ (<5.6 µg/L) | Ref. |  |
| 2^nd^ (5.7-8.9 µg/L) | 0.69 (0.56-0.85) | **<0.001** |
| 3^rd^ (9.0-14.1 µg/L) | 0.73 (0.59-0.90) | **0.003** |
| 4^th^ (>14.2 µg/L) | 0.52 (0.41-0.65) | **<0.001** |
| Statistically significant risk factors are reported in bold. Adjusted for cardiovascular risk factors, such as sex, LDL cholesterol, HDL cholesterol, BMI, lipid lowering therapy, blood pressure, diabetes mellitus, smoking, hsCRP and creatinine. Ref.: reference. | | |

**Suppl. Table 2**: Indices of inflammation according to vitamin B6 and HCY quartiles.

| Vitamin B6 quartiles (µg/L) | 1^st^  <5.6 | 2^nd^  5.7-8.9 | 3^rd^  9.0-14.1 | 4^th^  >14.2 | p-Value |
| --- | --- | --- | --- | --- | --- |
| hs-CRP  (mg/L) | 6.34  (1.15-38.20) | 3.83  (0.76-22.69) | 2.93  (0.60-14.64) | 1.81  (0.49-8.49) | **<0.001** |
| IL-6  (pg/mL) | 4.48  (1.42-16.02) | 3.29  (1.29-11.52) | 3.01  (1.14-9.68) | 2.30  (0.93-7.93) | **<0.001** |
| HCY quartiles (µmol/L) | 1^st^  <9.8 | 2^nd^  9.9-12.4 | 3^rd^  2.5-15.5 | 4^th^  >15.6 | p-Value |
| hs-CRP  (mg/L) | 2.86  (0.60-19.30) | 3.22  (0.64-20.92) | 3.58  (0.66-21.30) | 4.04  (0.82-25.70) | **<0.001** |
| IL-6  (pg/mL) | 2.80  (1.03-9.22) | 3.07  (1.16-10.27) | 3.20  (1.15-11.50) | 4.05  (1.40-14.36) | **<0.001** |

Variables are reported as median (10^th^-90^th^ percentiles). Statistically significant differences are reported in bold.

**Supplementary Fig. 1:** Flowchart of the LURIC study.

LURIC study

Ludwigshafen Risk and Cardiovascular Health study

RECRUITMENT started in 1997

*Prospective, monocentric, Cardiology in Ludwigshafen*

ENROLLMENT

INCLUSION CRITERIA:

German ancestry

Clinical stability

Availability of a coronary angiogram

Blood analyses of more than 3000 parameters (biochemical and genomics)

RECRUITMENT finished in 2002

*3316 patients*

10-YEARS FOLLOW-UP completed in 2012:

*2321 participants were still alive*

*995 participants had died (30%)*
